# Insights into the Karyotype Evolution of Charinidae, the Early-Diverging Clade of Whip Spiders (Arachnida: Amblypygi)

**DOI:** 10.3390/ani11113233

**Published:** 2021-11-12

**Authors:** Azucena Claudia Reyes Lerma, František Šťáhlavský, Michael Seiter, Leonela Zusel Carabajal Paladino, Klára Divišová, Martin Forman, Alexandr Sember, Jiří Král

**Affiliations:** 1 Laboratory of Arachnid Cytogenetics, Department of Genetics and Microbiology, Faculty of Science, Charles University, Viničná 5, 128 44 Prague, Czech Republic; areyes.lerma@gmail.com (A.C.R.L.); Dweep2@seznam.cz (K.D.); formivelkejpan@seznam.cz (M.F.); spider@natur.cuni.cz (J.K.); 2 Department of Zoology, Faculty of Science, Charles University, Viničná 7, 128 44 Prague, Czech Republic; stahlf@natur.cuni.cz; 3 Unit Integrative Zoology, Department of Evolutionary Biology, University of Vienna, Djerassiplatz 1, 1030 Vienna, Austria; michael.seiter@univie.ac.at; 4 Natural History Museum Vienna, 3. Zoology (Invertebrates), Burgring 7, 1010 Vienna, Austria; 5 Biology Centre of the Czech Academy of Sciences, Institute of Entomology, Branišovská 31, 370 05 České Budějovice, Czech Republic; leonela.carabajal@pirbright.ac.uk; 6 Arthropod Genetics Group, The Pirbright Institute, Ash Road, Pirbright, Woking GU24 0NF, UK; 7 Laboratory of Fish Genetics, Institute of Animal Physiology and Genetics, Czech Academy of Sciences, Rumburská 89, 277 21 Liběchov, Czech Republic

**Keywords:** *Charinus*, chromosome fusion, fluorescence *in situ* hybridization, heterochromatin, nucleolar organizer region, *Sarax*, telomere

## Abstract

**Simple Summary:**

Whip spiders (Amblypygi) are spectacular arachnids characterized by powerful raptorial pedipalps and exceptionally elongated forelegs. Although cytogenetic data from amblypygids might be, given their phylogenetic position, important for the reconstruction of arachnid ka-ryotype evolution, cytogenetics of this order is still largely understudied. Here, we applied conventional and molecular cytogenetics to describe the karyotype patterns in Charinidae—the family placed almost at the base of amblypygid phylogeny, thereby providing insights into the ancestral amblypygid karyotype traits. We surveyed four *Charinus* and five *Sarax* species and found a wide range of diploid chromosome numbers (2*n*) in both genera. Representatives with lower 2*n* possessed generally a higher proportion of biarmed (metacentric and submetacentric) chromosomes. Our results indicate the important roles of chromosome fusions and pericentric inversions in the karyotype differentiation of Charinidae, similarly to that suggested previously for neoamblypygids. Our data, gathered from C-banding, fluorescent banding, and chromosomal mapping of ribosomal DNA and telomeric repeats, bring evidence for the action of these rearrangements and suggest the general trajectory towards 2*n* reduction from ancestral high chromosome counts. However, we cannot rule out the contribution of chromosome fissions.

**Abstract:**

Whip spiders (Amblypygi) represent an ancient order of tetrapulmonate arachnids with a low diversity. Their cytogenetic data are confined to only a few reports. Here, we analyzed the family Charinidae, a lineage almost at the base of the amblypygids, providing an insight into the ancestral traits and basic trajectories of amblypygid karyotype evolution. We performed Giemsa staining, selected banding techniques, and detected 18S ribosomal DNA and telomeric repeats by fluorescence *in situ* hybridization in four *Charinus* and five *Sarax* species. Both genera exhibit a wide range of diploid chromosome numbers (2*n* = 42–76 and 22–74 for *Charinus* and *Sarax*, respectively). The 2*n* reduction was accompanied by an increase of proportion of biarmed elements. We further revealed a single NOR site (probably an ancestral condition for charinids), the presence of a (TTAGG)*_n_* telomeric motif localized mostly at the chromosome ends, and an absence of heteromorphic sex chromosomes. Our data collectively suggest a high pace of karyotype repatterning in amblypygids, with probably a high ancestral 2*n* and its subsequent gradual reduction by fusions, and the action of pericentric inversions, similarly to what has been proposed for neoamblypygids. The possible contribution of fissions to charinid karyotype repatterning, however, cannot be fully ruled out.

## 1. Introduction

Whip spiders (Amblypygi) represent an ancient arachnid order of tropical and subtropical nocturnal predators. They inhabit diverse biotopes of all continents, except for Antarctica [1]. Their distribution area ranges from the Italian port Trieste (the northernmost record) [2] to the Republic of South Africa (the southernmost record) [1]. Amblypygids are characterized by a dorsoventrally flattened body, raptorial pedipalps, and extremely elongated, antenniform first pair of legs [1,3]. Whip spiders seem to display rather low diversity, though their fossil records date back to the Upper Carboniferous [1,4]. Recent studies, however, suggest that the amblypygid diversity might be considerably larger, but its cryptic nature hampers its thorough characterization by most of the current approaches [5,6,7]. Amblypygids are classified into two suborders, namely the Paleoamblypygi, represented by the monotypic genus *Paracharon*, and the Euamblypygi, which includes four families (Charinidae, Charontidae, Phrynidae, and Phrynichidae) encompassing about 220 extant species placed in 17 genera [3,8,9,10,11,12]. Amblypygids, whip scorpions (Uropygi), schizomids (Schizomida), and spiders (Araneae) form the large clade Tetrapulmonata. This lineage is well supported in recent schemes of arachnid phylogeny based on molecular data [13,14,15].

Cytogenetic data on amblypygids are scarce, with the first record dating back to a study of Millot and Tuzet [16], which provides information on the diploid chromosome number (2*n*) and sex chromosome constitution in *Sarax sarawakensis* (Charinidae). The 2*n* and telomere sequence mapping was reported for *Damon medius* (Phrynichidae) by Vítková et al. [17]. Later, Paula-Neto et al. [18] shed light on the karyotype of *Heterophrynus longicornis* (Phrynidae), including the distribution of heterochromatin and the number and position of nucleolar organizer regions (NORs). Recently, the karyotype data of one *Phrynus* and six *Paraphrynus* species (Phrynidae) were reported by Seiter et al. [7], who evidenced a wide range of 2*n* (from 24 to 68). Karyotype differentiation of *Paraphrynus* involved centric fusions and pericentric inversions.

In the present work, we aim to elucidate karyotype differentiation in the family Charinidae, the earliest-diverging lineage among euamblypygids. Charinids consist of three genera (*Charinus*, *Sarax*, and *Weygoldtia*) and 132 species [10,11,19,20,21]. The charinid lineage is sister to the remainder of the euamblypygids, which form the so-called neoamblypygid clade [10,22]. It is presumed that the radiation of charinids and neoamblypygids was initiated before the fragmentation of the Pangean and Gondwanian supercontinents, which might explain their recent distribution [1,22]. Due to their basal phylogenetic position within whip spiders, charinids are an important target for analyses of karyotype evolution in whip spiders and Tetrapulmonata. We conducted both conventional (Giemsa staining, C and fluorescent banding, silver staining of NORs) and molecular cytogenetic analyses [chromosomal mapping of (TTAGG)*_n_* telomeric sequence and 18S ribosomal DNA (rDNA) sites by fluorescence *in situ* hybridization (FISH)].

## 2. Materials and Methods

### 2.1. Individuals

Twenty-one individuals belonging to four *Charinus* and five *Sarax* species were analyzed (for details, see Table 1). Voucher specimens are mostly deposited in the Laboratory of Arachnid Cytogenetics, Department of Genetics and Microbiology (Faculty of Science, Charles University, Prague). Specimens of *S. seychellarum* are placed in the collection of the Department of Zoology (Faculty of Science, Charles University, Prague) and the samples of *C. pescotti* are deposited in the collection of the Western Australian Museum, Perth (Australia).

### 2.2. Chromosome Preparations and Evaluation of Karyotypes

Mitotic and meiotic chromosomes were obtained from gonadal tissue of preferentially adult or subadult specimens using a spreading technique described originally for butterflies by Traut [23], with several modifications (detailed in [24]). Briefly, the tissue was hypotonized in 0.075 M KCl for 20 min, and then fixed in three rounds of freshly prepared ethanol: acetic acid (3:1; *v*/*v*) for 6, 10, and 20 min. A piece of fixed gonad was suspended in a drop of 60% acetic acid placed on a clean slide. The cell suspension was spread on a slide placed on a histological plate heated to 40 °C, using a tungsten needle. The chromosome preparations were subsequently stained in 5% Giemsa solution in Sörensen buffer (pH 6.8) for 30 min. To evaluate the chromosome morphology and to construct the karyotypes, at least five mitotic metaphases (*C. dominicanus*, *C. pescotti*, *S*. aff. *batuensis*, *S. seychellarum*) or meiotic metaphases II (*C. cavernicolus*, *C. neocaledonicus*, *S. huberi*, *Sarax* sp.) were analyzed. In *S. ioanniticus*, the quality of metaphase spreads only permitted the identification of the 2*n* and an approximate determination of chromosome morphology. We obtained just a limited number of chromosome plates due to the small size of the gonads and the low number of specimens collected. Therefore, selected techniques of chromosome banding and molecular cytogenetics could be applied only to some species. The sequential use of the slides for more techniques was met with varied success (see Table 1).

Classification of chromosome morphology was based on the position of the centromere according to Levan et al. [25], but modified as metacentric (m), submetacentric (sm), subtelocentric (st), and acrocentric (a). To calculate the FN value (number of chromosome arms, fundamental number [26]), metacentric and submetacentric chromosomes were scored as biarmed, while subtelocentric and acrocentric chromosomes were scored as monoarmed.

### 2.3. Chromosome Banding Techniques

The protocol for chromosome preparation was modified for C-banding. Specifically, the tissue was fixed in methanol: acetic acid (3:1), and the temperature of the histological plate was reduced to 35 °C for better preservation of the chromatin. The distribution of the constitutive heterochromatin was analyzed using the C-banding method following Sumner [27]. Preparations were dried and stained with 5% Giemsa in Sörensen buffer (pH 6.8) for 75 min. In *C. pescotti*, the chromosomes were stained with 4′,6-diamidino-2-phenolindole (DAPI) (FluoroshieldTM; Sigma-Aldrich, St. Louis, MO, USA) and the pictures were were inverted for better contrast. This protocol modification provides a better resolution of the C-bands and, hence, allows visualization of the tiny blocks of constitutive heterochromatin. Although it has already been repeatedly used in some other arachnids (e.g., [28,29]), we are aware that it might, on the other hand, underestimate GC-rich heterochromatin blocks.

The composition of constitutive heterochromatin was determined in *C. neocaledonicus* using the GC-specific fluorochrome chromomycin A_3_ (CMA_3_) and the AT-specific fluorochrome DAPI (both Sigma-Aldrich) following the method of Sola et al. [30] and Mayr et al. [31].

Silver staining of transcriptionally active NORs [32,33] was performed according to Dolejš et al. [24], with a modified staining time ranging from 5 to 7 min.

### 2.4. Telomeric FISH

A telomeric probe corresponding to the insect telomeric motif (TTAGG)*_n_* was generated by non-template PCR according to Ijdo et al. [34], following the protocol modification of Sahara et al. [35]. The probe was labeled with biotin-14-dUTP by nick translation using a Nick Translation Kit (Abbott Molecular, IL, USA). The FISH was carried out according to the protocol described above for rDNA FISH, and the probe was also detected with Cy3-conjugated streptavidin. The chromosomes were counterstained with DAPI (Fluoroshield™; Sigma-Aldrich).

### 2.5. Detection of 18S rDNA by FISH

The 18S rDNA probe was obtained from the spider *Dysdera erythrina* (Dysderidae) by polymerase chain reaction (PCR) amplification using primers 18SF: 5′-CGAGCGCTTTTATTAGACCA-3′ and 18SR: 5′-GGTTCACCTACGGAAACCTT-3′ (Generi Biotech, Hradec Králové, Czech Republic) [36]. The probe was labeled with biotin-14-dUTP using the BioNick™ DNA Labeling System (Invitrogen Life Technologies, San Diego, CA, USA) according to the manufacturer’s instructions. The FISH technique followed Fuková et al. [37]. In brief, after dehydration (70, 80, and 96% ethanol series, 1 min each at room temperature; RT), the slides were treated with RNase A (200 ng/μL in 2× SSC; 60 min, 37 °C). The chromosomes were denatured in 70% formamide in 2× SSC (pH 7.0) for 3.5 min at 68 °C, and then dehydrated through a 70% (−20 °C), 80%, and 96% (RT) ethanol series. The hybridization mixture (10 µL per slide; containing 20 ng of biotin-labeled 18S rDNA probe, 25 µg of sonicated salmon sperm DNA, 10% dextran sulphate, and 50% formamide in 2× SSC) was denatured at 90 °C (5 min), cooled on ice, and applied onto the slides. Hybridization took place overnight at 37 °C in a dark humid chamber. Post-hybridization washes were carried out three times in 50% formamide in 2× SSC (46 °C, 5 min each) and three times in 0.1× SSC (62 °C, 5 min each). A blocking mixture of 500 μL of 2.5% BSA (GERBU Biotechnik GmbH, Heidelberg, Germany) in 4× SSC was applied on each slide (RT, 20 min). The probe was detected with Cy3-conjugated streptavidin (Invitrogen Life Technologies), with additional signal enhancement by treatment with biotinylated anti-streptavidin (Vector Laboratories, Burlingame, CA, USA) and a second round of streptavidin–Cy3 detection according to Fuková et al. [37]. The preparations were counterstained with 0.5 µg/mL DAPI in PBS containing 1% Triton X-100, mounted in antifade based on 1,4-diazabicyclo(2.2.2)-octane (DABCO; Sigma–Aldrich) (for composition, see [38]), and covered with a glass coverslip.

### 2.6. Microscopy and Image Analysis

Standard preparations stained by Giemsa were inspected under a BX 50 microscope (Olympus, Tokyo, Japan) and images were photographed under immersion objective 100× using a DP 71 CCD camera (Olympus). Pictures from FISH and C-banding were captured by an IX81 microscope (Olympus) equipped with an ORCA-AG CCD camera (Hamamatsu Photonics, Hamamatsu, Japan). The digital images from the FISH and fluorescent banding were pseudocolored (red for Cy3 and CMA_3_, green or blue for DAPI) and superimposed with the Cell^R software (Olympus Soft Imaging Solutions GmbH, Muenster, Germany). Fluorescent banding was evaluated under immersion objective 100× using a Provis AX70 Olympus microscope with an appropriate fluorescence filter set. Images were photographed by a black and white DP30W CCD Olympus camera for each fluorescent dye using Olympus Acquisition Software. Karyotypes were arranged in Corel PHOTO-PAINT X4 software (Corel, Ottawa, ON, Canada). Composed images were optimized and arranged using Adobe Photoshop CS6 (Adobe Systems, San Jose, CA, USA).

## 3. Results

### 3.1. Karyotype

**Figure 1:**
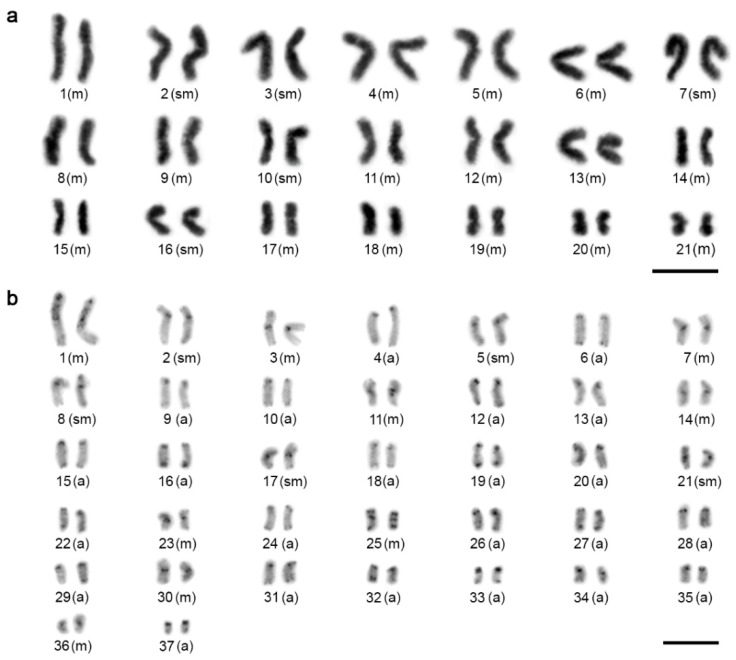
Male karyotypes of *Charinus* species I, constructed from mitotic metaphases. (**a**) *C. dominicanus* (Giemsa-stained, without banding) (2*n* = 42). (**b**) *C. pescotti* (C-banding; chromosomes stained with DAPI, inverted picture) (2*n* = 74). Abbreviations: m = metacentric chromosome, sm = submetacentric chromosome, st = subtelocentric chromosome, a = acrocentric chromosome. Scale bar = 10 μm.

**Figure 2:**
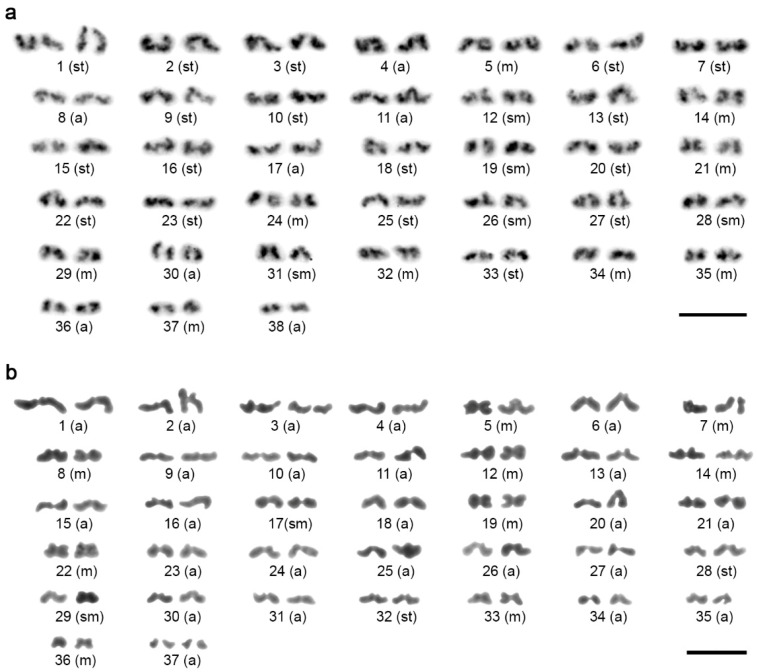
Male karyotypes of *Charinus* species II, Giemsa-stained. Karyotypes were constructed from two sister metaphases II. (**a**) *C. cavernicolus* (2*n* = 76). (**b**) *C. neocaledonicus* (2*n* = 74). Abbreviations: m = metacentric chromosome, sm = submetacentric chromosome, st = subtelocentric chromosome, a = acrocentric chromosome. Scale bar = 10 μm.

Diploid chromosome numbers of *Charinus* ranged widely—from 42 to 76, with FN ranging from 84 to 104 (Figure 1 and Figure 2). There were two karyotype patterns within *Charinus*, which differed considerably by 2*n* and chromosome morphology. While a single species, *C. dominicanus*, exhibited a lower 2*n* (42, FN = 84) and its karyotype was composed of biarmed elements only (32m + 10sm; Figure 1a), the three remaining species displayed high 2*n* and a predominance of monoarmed chromosomes: *C. pescotti* (2*n* = 74, FN = 102; 18m + 10sm + 46a; Figure 1b), *C. cavernicolus* (2*n* = 76, FN = 104; 10m + 18sm + 36st + 12a; Figure 2a), and *C. neocaledonicus* (2*n* = 74, FN = 96; 18m + 4sm + 4st + 48a; Figure 2b).

**Figure 3:**
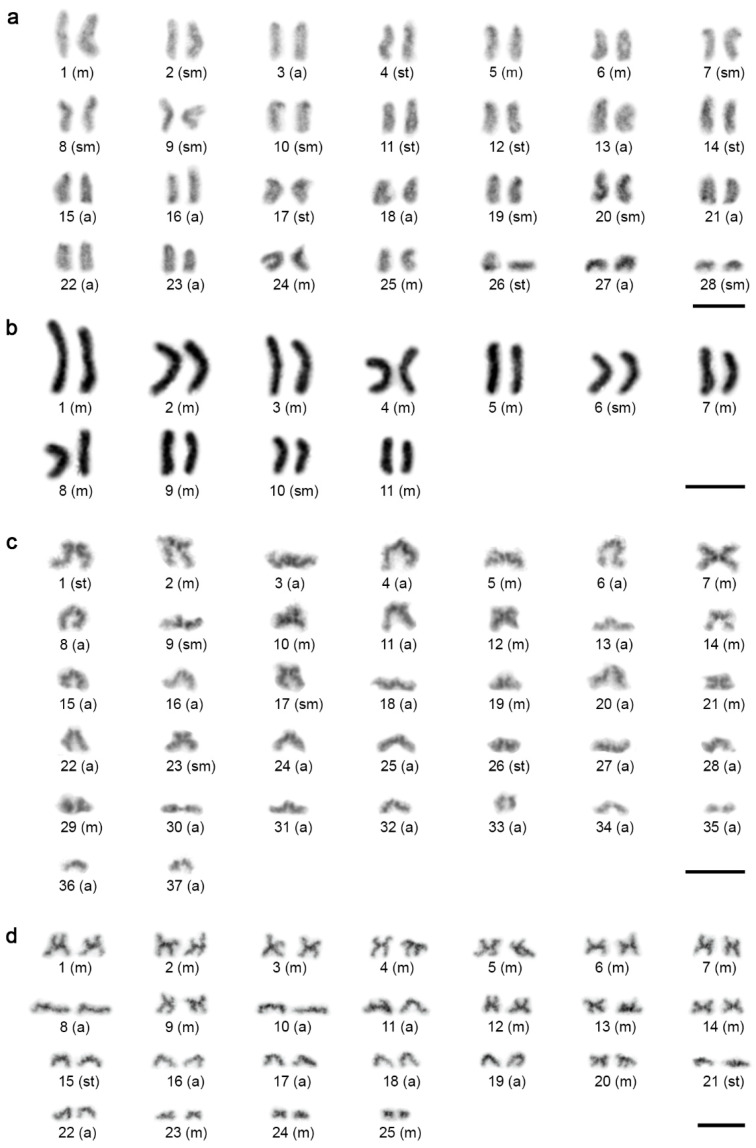
Male and female karyotypes of *Sarax* species, Giemsa-stained. Karyotypes were constructed from mitotic metaphase (**a**,**b**), metaphase II (**c**), or two sister metaphases II (**d**). (**a**) *S*. aff. *batuensis*, male (2*n* = 56). (**b**) *S. seychellarum*, female (2*n* = 22). (**c**) *Sarax* sp., male (*n* = 37), haploid karyotype. (**d**) *S. huberi*, male (2*n* = 50). Abbreviations: m = metacentric chromosome, sm = submetacentric chromosome, st = subtelocentric chromosome, a = acrocentric chromosome. Scale bar = 10 μm.

**Figure 4:**
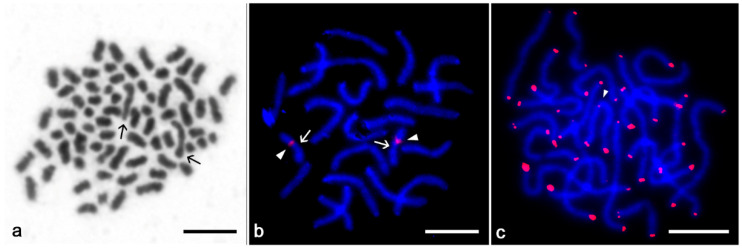
Chromosomes of *Sarax* after Giemsa staining (**a**) and mapping of repetitive DNAs by FISH (**b**,**c**). (**a**,**b**) Mitotic metaphases; (**c**) mitotic prometaphase. (**a**) *S. ioanniticus*, female. Chromosomes of the largest pair (arrows) differ by degree of condensation from the rest of the complement. (**b**) *S. seychellarum*, female. Chromosomal mapping of 18S rDNA clusters (red signals, arrowheads). Centromeres are marked by arrows. Chromosomes counterstained with DAPI (blue). (**c**) *S. seychellarum*, male. Chromosomal mapping of telomeric motif (TTAGG)*_n_* (red signals). Arrowhead points to the interstitial telomeric site. Chromosomes counterstained with DAPI (blue). Scale bar = 10 μm.

We found even wider karyotype diversity in *Sarax*, with 2*n* ranging from 22 to 74 chromosomes (Figure 3 and Figure 4a). The karyotype of *Sarax* sp. comprised 74 chromosomes (FN = 98), with a predominance of monoarmed elements (18m + 6sm + 4st + 46a; Figure 3c), and female mitotic metaphases of *S. ioanniticus* displayed 72 predominantly monoarmed chromosomes (Figure 4a; more detailed analysis of chromosome morphology was impossible due to the high degree of chromosome condensation, with primary constrictions not clearly visible). In striking contrast, the karyotype of *S. seychellarum* was composed of 22 biarmed chromosomes (FN = 44; 18m + 4sm; Figure 3b). The karyotypes of the remaining two *Sarax* species displayed intermediary patterns, with 2*n* equal to 50 or 56 chromosomes and only slight predominance of biarmed chromosomes: *S*. aff. *batuensis* (2*n* = 56, FN = 82; 10m + 16sm + 12st + 18a; Figure 3a) and *S. huberi* (2*n* = 50, FN = 80; 30m + 4st + 16a; Figure 3d).

Chromosomes of charinids decreased gradually in size, except for the longest chromosome pair of *S. ioanniticus*. During oogonial mitosis, this pair exhibited a different degree of condensation than the other chromosomes in the set (Figure 4a). Morphologically differentiated sex chromosomes were not observed in any of the species under study.

### 3.2. Distribution and Composition of Constitutive Heterochromatin

**Figure 5:**
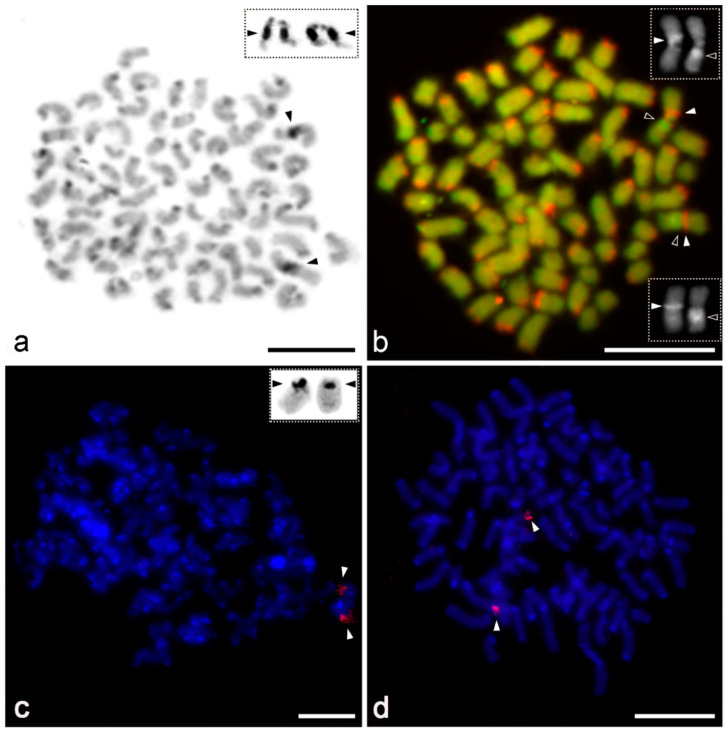
Male chromosomes of *Charinus* species after chromosome banding techniques (**a**,**b**) or 18S rDNA FISH (**c**,**d**). (**a**,**b**,**d**) Mitotic metaphase; (**c**) meiotic prophase I, early diplotene. (**a**–**c**) *C. neocaledonicus.* (**a**) C-banding (Giemsa-stained). Note a prominent (peri)centromeric block of constitutive heterochromatin on chromosomes of a large chromosome pair (arrowheads). Inset depicts the same chromosome pair from C-banded meiotic metaphase II pointing to its acrocentric morphology. (**b**) Fluorescent banding with two fluorochromes, GC-specific CMA_3_ and AT-specific DAPI. For better contrast, pictures were pseudocolored in red for CMA_3_ and green for DAPI. Note a large chromosome pair with a huge block of (peri)centromeric heterochromatin formed by two segments differing in affinity to applied fluorochromes; CMA_3_^+^ band (full arrow) vs. DAPI^+^ band (empty arrow). Boxes: CMA_3_ (left) and DAPI staining (right) of particular chromosomes of the pair. (**c**) Chromosomal mapping of 18S rDNA clusters (red signals, arrowheads). Chromosomes of one bivalent contain the cluster. Chromosomes were counterstained with DAPI (blue). Inset depicts NOR-bearing chromosome pair after silver staining (arrowheads point to the terminal NOR). (**d**) *C. pescotti*. Detection of 18S rDNA clusters (red signals, arrowheads), chromosomes counterstained with DAPI (blue). Chromosomes of one pair contain a terminal cluster. Scale bar = 10 μm.

C-banding was performed in two *Charinus* species—*C. neocaledonicus* (Figure 5a) and *C. pescotti* (Figure 1b). In both species, the blocks of constitutive heterochromatin were mostly confined to the terminal parts of the chromosomes. The (peri)centromeric region of all monoarmed chromosomes was formed by heterochromatin. Moreover, acrocentric pairs nos 6, 16, 19, 22, 27, 32, and 33 displayed an additional band at the end of their long arms. In *C. pescotti*, approximately one-third of the chromosomes possessed constitutive heterochromatin in their middle or subterminal part. These blocks corresponded to the (peri)centromeric region of biarmed chromosomes (Figure 1b). The metacentric pair no. 25 exhibited (peri)centromeric heterochromatin and two terminal bands, each at one chromosome arm. A remarkable interstitial block of constitutive heterochromatin was identified in one acrocentric pair (possibly pair no. 1) of *C. neocaledonicus* (Figure 5a). Additional analysis by fluorescent banding revealed that this block is composed of two segments differing considerably in their base composition (Figure 5b). While one region was GC-rich (i.e., CMA_3_-positive), the other one was AT-rich (i.e., DAPI-positive). CMA_3_-positive signals were also localized at one end of most chromosomes, while DAPI-positive regions were less pronounced. They were often placed at the opposite chromosome ends.

### 3.3. Chromosomal Distribution of Telomeric Repeats

The FISH with the telomeric probe (TTAGG)*_n_* was performed only in *S. seychellarum*. Since this species exhibits the lowest 2*n* in our sampling, it is the one with a possible high incidence of chromosome rearrangements, herein particularly fusions, which may produce interstitial telomeric sites (ITSs). The probe revealed signals at the ends of all chromosomes, and a single ITS on one chromosome (Figure 4c).

### 3.4. Distribution of 18S rDNA/NOR

A single NOR-bearing chromosome pair was detected in *C. neocaledonicus* (Figure 5c), *C. pescotti* (Figure 5d), and *C. seychellarum* (Figure 4b). In *C. neocaledonicus* and *C. pescotti*, the NOR occupied the terminal chromosomal region (Figure 5c,d). In contrast, the NOR of *C. seychellarum* was placed interstitially, more specifically, in the middle of one arm of the smallest metacentric chromosome pair (no. 11) (Figure 4b).

## 4. Discussion

### 4.1. Patterns of Karyotype Differentiation

The cytogenetic features of amblypygids are highly underexplored. Apart from an early report on the charinid *S. sarawakensis* [16], they are confined to two derived families, Phrynidae and Phrynichidae [7,17,18]. With nine newly analyzed species, the present study increased the number of cytogenetically analyzed amblypygids to 18.

**Figure 6:**
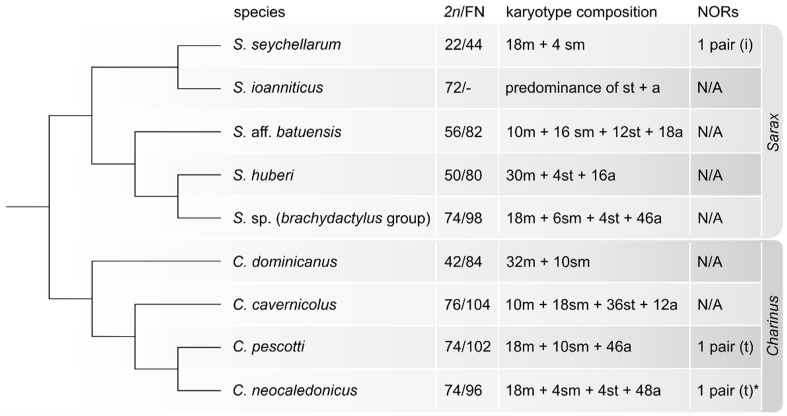
Charinidae, karyotype features, including number and position of NOR loci. Mapped on a cladogram constructed based on the recent molecular phylogeny of Miranda et al. [10]. Abbreviations: a = acrocentric chromosome, FN = fundamental number, m = metacentric chromosome, N/A = not analyzed, I = interstitial position, NORs = distribution of nucleolar organizer regions, sm = submetacentric chromosome, st = subtelocentric chromosome, t = terminal position. * In *C. neocaledonicus*, the NOR distribution has been assessed by both silver staining and rDNA FISH techniques.On the contrary, in two remaining species, only rDNA FISH has been carried out. We did not include the karyotype data of Millot and Tuzet [16] on *Sarax sarawakensis.* The authors indicated 2*n* = 25 for males, including a single X chromosome (the sex chromosome system ♂X0/♀XX). The graphical documentation of results presented by these authors is not sufficient to support their statements.

Available cytogenetic reports on amblypygids point to a striking diversity in 2*n* [7,16,17,18] and chromosome morphology [7]. Moreover, our results on Charinidae, an early diverging amblypygid lineage, showed a wide range of 2*n* in *Sarax* (2*n* = 22–74) and *Charinus* (2*n* = 42–76). The karyotypes with higher 2*n* were composed predominantly of mono-armed chromosomes, while the karyotypes with low 2*n* consisted exclusively of biarmed elements. These observations suggest the major role of chromosome fusions or fissions in the karyotype evolution of charinids. To infer the direction of this process and, hence, reconstruct the ancestral charinid karyotype, the obtained karyotype patterns were plotted onto the recent charinid phylogenetic hypothesis [10], as seen in Figure 6.

Given the considerable karyotype diversity in both analyzed genera, we are currently unable to propose a coherent scenario regarding the main trajectory of the karyotype reshuffling. We cannot exclude similar mechanisms of karyotype evolution (i.e., centric fusions and pericentric inversions) as described in the derived amblypygid lineage Phrynidae, wherein the comparably wide range of 2*n* (24–68) has been reported [7]. While the available data do not allow a conclusive assessment about ancestral karyotype pattern in charinids and amblypygids in general, there are some indications supporting high ancestral 2*n*. We found very similar karyotypes in *Charinus* and *Sarax* with high chromosome counts (*C. cavernicolus*, 2*n* = 76; *C. pescotti* and *C. neocaledonicus*, 2*n* = 74; *S. ioanniticus*, 2*n* = 72 and *Sarax* sp., 2*n* = 74). These species display a predominance of monoarmed chromosomes. It is much less probable that a series of independent chromosome fissions would convergently lead to such a similar chromosome count; hence, it is more likely that the process took the opposite trajectory, which has led to the reduction in high ancestral 2*n*. Unfortunately, we have no information about the degree of conserved synteny among the charinid karyotypes and, therefore, the real interspecific karyotype differences remain unknown. We may only suppose (bearing in mind also the supportive results from other methods, see below) that chromosomal fusions leading to the reduction in high ancestral 2*n* represent one of the main mechanisms of karyotype differentiation in Charinidae. This notion is corroborated by the comparably high 2*n* in two derived amblypygid lineages, specifically in *Damon medius* (2*n* = 70; Phrynichidae) [17] and three species belonging to Phrynidae (*Heterophrynus longicornis*, 2*n* = 66; *Paraphrynus robustus*, 2*n* = 64; *Phrynus marginemaculatus*, 2*n* = 68) [7,18]. The high ancestral 2*n* of amblypygids may also be consistent with the recently proposed theory regarding the whole genome duplication in the common ancestor of scorpions and tetrapulmonate arachnids [39,40,41]. The conception of high 2*n* in ancestral tetrapulmonates is supported by high chromosome counts in most basal spiders, mesothelids [42]. However, our current data do not fully rule out the opposite scenario, i.e., low ancestral 2*n* in Charinidae and its subsequent convergent elevation within *Sarax* and *Charinus* by fissions. This alternative hypothesis is consistent with the basal phylogenetic position of *C. dominicanus*, a species with low 2*n* (Figure 6).

In Phrynidae, the main underlying mechanism for karyotype change was proposed to be centric fusion [7]. Among our surveyed charinids, we found a putative footprint of a fusion event in the large chromosome pair of *C. neocaledonicus*. This pair contains the two large adjacent segments of constitutive heterochromatin, which differ considerably by their sequence composition (one was AT-rich, while the other one was GC-rich). Differential enrichment in AT vs. GC base pairs in these segments suggests that they belonged originally to two separate chromosomes, which fused during evolution. A similar situation was observed in animal taxa known for their high rate of chromosome fusions (e.g., [43]). In *C. neocaledonicus*, centromere-to-telomere fusion (i.e., tandem fusion) of two smaller monoarmed chromosomes is likely, as (i) the AT- and GC-rich heterochromatin usually occupy the opposite ends of the chromosomes in this species, and (ii) the pair containing two tandemly arranged blocks is acrocentric. There are no data from other amblypygids concerning the distribution of AT- and GC-rich heterochromatin. Among other arachnids, diverse patterns of GC-rich heterochromatin have been recorded in spiders and scorpions, from a scattered distribution of many GC-rich bands throughout the chromosome complement to terminal bands localized on several chromosomes (e.g., [44,45,46]). Another indication of the action of fusions in the charinid karyotype evolution is the presence of an internal telomeric site (see below).

Comparably to phrynids [7], the elevated number of biarmed chromosomes in charinids with lower 2*n* cannot be fully explained by only a series of fusions. If this were the case, FN would be maintained roughly the same among the species and the number of biarmed elements would increase proportionally to the decrease in 2*n* [26]. The conside-rable deviation in FN found in charinids suggests that their karyotypes were also shaped by rearrangements, which changed the chromosome morphology without affecting the 2*n*. For example, the karyotypes of *C. neocaledonicus* and *C. pescotti* are composed of the same number of chromosomes, but these species display different proportions of monoarmed and biarmed chromosomes, and hence different FN (96 for *C. neocaledonicus* and 102 for *C. pescotti*). Our rDNA data (see below) strongly favor the action of inversions, although the involvement of other mechanisms leading to centromeric shift (e.g., [47]) cannot be excluded.

As stated above, amblypygids exhibit a considerable karyotype differentiation. Besides amblypygids, a high diversity of karyotypes has also been found in other arachnid orders, namely in spiders (2*n* = 5–152; [48,49,50]), scorpions (2*n* = 5–186; [51,52]), harvestmen (2*n* = 10–109; [53,54]), and pseudoscorpions (2*n* = 7–143; [55,56]). Compared to other arachnids, the amblypygid radiation, which took place before the collapse of Pangea and Gondwana, led to the occurrence of amblypygids in almost all continents [22]—a condition also notable in charinids [19,20,21]. This process could expose the amblypygid taxa to various selective pressures associated with the need for adaptation to diverse habitats and ecolo-gical niches. Together with the considerable fragmentation of amblypygid populations, these conditions have contributed to the karyotype diversification of this arachnid group. The regions of constitutive heterochromatin may considerably facilitate the incidence of chromosome rearrangements [57] and our data in *C. neocaledonicus* suggest that this might also be the case in charinids. In turn, although not necessarily linked to speciation (e.g., [58]), chromosomal rearrangements may contribute to the emergence of postzygotic reproductive barriers between conspecific populations [59,60,61,62,63] and may lead to genetic innovations (e.g., by altering gene expression, establishment of tight linkage between alleles under positive selection), which may be favorable, for example, for local adaptation [63,64,65,66]. Alternatively, chromosome rearrangements might be fixed by genetic drift [59].

We did not observe heteromorphic (i.e., morphologically differentiated) sex chromosomes in any of the studied charinids, which is in line with the observations in other ka-ryotyped amblypygids [7,18], except for charinid *S. sarawakensis* [16], where the authors proposed the ♂X0/♀XX sex chromosome system (2*n* = 25 in males). However, the pre-sence of an X0 system in *S. sarawakensis* is unlikely, as we did not find heteromorphic sex chromosomes in other *Sarax* species. The lack of heteromorphic sex chromosomes in amblypygids, nonetheless, does not rule out the possibility that homomorphic sex chromosomes (i.e., those without morphological differentiation) might be present in them. Given that mostly only males (in seven out of nine species) provided chromosome plates, one cannot exclude female heterogamety with morphologically differentiated sex chromosomes in whip spiders. Heteromorphic sex chromosomes occur in two tetrapulmonate groups, spiders [48,67,68,69] and uropygids [70]. These orders exhibit male heterogamety. Bearing in mind that sex chromosomes of spiders exhibit a specific behavior, including a different condensation both in male and female germlines [71], we suppose that a large homomorphic chromosome pair exhibiting a distinct condensation pattern in the oogonial mitoses of *S. ioanniticus* females might potentially represent sex chromosomes. This hypothesis is amenable for experimental testing in further studies.

### 4.2. Distribution of rDNA and Telomeric Sequences

The major rDNA cluster (45S rDNA) is by far the most utilized molecular chromosomal marker across diverse eukaryotic organisms, and especially in non-model groups [72,73,74]. The major rDNA cluster visualized either by 18S or 28S rDNA probes has already been localized in some spiders [36,46,73,75,76,77], scorpions [45,76,78,79,80,81,82], pseudoscorpions [83], harvestmen [84,85,86,87], ticks [73,88,89], and an amblypygid [18]. Mapping of this cluster often revealed a single NOR site, which is probably an ancestral pattern in arachnids [36]. The number of NOR/rDNA loci has increased in some arachnid clades during their evolution [76,77,81,87]. In haplogyne spiders, these loci are also frequently placed on sex chromosomes [77,90]. When stable distribution patterns for rDNA loci within a certain lineage are used as markers in the phylogenetic context, they may largely contribute to the interpretation of possible karyotype rearrangements (e.g., [84]). All three charinids examined in the present study possess a single pair of NOR-bearing chromosomes, thus implying a possible ancestral condition for the whole amblypygid clade. While in two *Charinus* species (*C. neocaledonicus* and *C. pescotti*), the NOR occupies the terminal position on a chromosome pair, in a single surveyed *Sarax* species (*S. seychellarum*), the NOR is placed interstitially in the middle of one arm of the smallest metacentric chromosome pair. The most parsimonious explanation for the observed pattern is that a paracentric inversion shifted the NOR from the terminal location to the interstitial one in this species. Therefore, our data suggest that the major rDNA cluster might be a useful cytogenetic marker for the determination of mechanisms of karyotype repatterning in amblypygids.

According to our data, DNA molecules of charinids are terminated by the so-called insect telomeric sequence (TTAGG)*_n_*, which is ancestral for arthropods [17]. This motif has been identified in many arachnid orders [17,35,45,78,79,89,91], including a whip spider of the family Phrynichidae, *Damon medius* [17]. While the same telomeric motif is usually shared by taxa on higher taxonomic levels, exceptions do occur [92,93,94,95], including arachnids [17]. The presence of insect telomeric sequence, both in early-branching charinids and in a representative of phrynichids belonging to neoamblypygids, suggests that this motif is ancestral in amblypygids. This conclusion is in line with the wide distribution of this motif in arachnids, except for spiders [17]. Besides terminal (TTAGG)*_n_* sequences, we also found one ITS in *S. seychellarum*. This ITS could have arisen by chromosome rearrangement (e.g., [96,97]; for reviews, see [98,99]). *S. seychellarum* stands out from our sampling by possessing the lowest 2*n* (22 chromosomes), with the karyotype being composed of biarmed elements only. Therefore, the observed ITS might represent a hallmark of a previous chromosome fusion or translocation. The presence of ITS on only one of the homologues is puzzling but it might reflect the heterozygous constitution resulting from a different degree of sequence elimination/degeneration from the interstitial site. Besides chromosome rearrangement, the telomeric repeats might also be translocated to the interstitial region via transposition or during double-strand break repair processes [98,99].

## 5. Conclusions

Our cytogenetic survey in the early-branching amblypygid family Charinidae provides important clues about the pace and trajectories of karyotype differentiation in amblypygids and in tetrapulmonates in general. The data corroborate the findings from the derived lineage Phrynidae in the way that amblypygids possess highly variable 2*n* and chromosome morphology shaped mostly by fusions and inversions. While we suppose the general direction of karyotype change towards a reduction in 2*n* via fusions in amblypygids, and some of our findings support this view, larger sampling, including representatives of other amblypygid families, is needed to clarify this hypothesis.

## Figures and Tables

**Table 1:** Studied species, number of analyzed individuals and their sex, collection sites, and methods applied.

Species	N	Source or Locality	Methods
*Charinus cavernicolus* Weygoldt, 2006	2♂, 1 juv	Grottes de Koumac, Koumac, New Caledonia	G, K
*C. dominicanus* Armas & Gonzáles Perez, 2001	1♂	Los Charcos, San Rafael, La Ciénaga Municipality, Barahona Province, Dominican Republic	G, K
*C. neocaledonicus* Simon in Kraepelin, 1895	5♂	Mount Coghis, close to Noumea, New Caledonia	G, K, C, F, Ag, rDNA
*C. pescotti* Dunn, 1949	3♂	Mossman river Queensland, Australia	G, K, C, rDNA
*Sarax* aff. *batuensis* Roewer, 1962	1♂	Malaysia breeding	G, K
*S. huberi* Seiter et al., 2015	1♂	surroundings of the Busay cave, Moalboal, Cebu Island, Philippines	G, K
*S. ioanniticus* Kritscher, 1959	3♀	casemates of the fortress, Rhodos, Rhodos Island, Greece	G
*S. seychellarum* Kraepelin, 1898	1♂, 2♀	Mahé Island, Republic of Seychelles	G, K, rDNA, T
*Sarax* sp. *	1♂	Malaysia breeding	G, K

* Undetermined species belonging to *Sarax brachydactylus* group. Abbreviations: N = number of analyzed individuals, juv = unsexed juvenile, Ag = silver staining of nucleolar organizer regions, C = C-banding, F = fluorescent banding, G = Giemsa staining, K = karyotyping, rDNA = FISH with 18S rDNA probe, T = telomeric FISH.

## Data Availability

The data presented in this study are available upon request from the corresponding author.
